# Timed Maternal Melatonin Treatment Reverses Circadian Disruption of the Fetal Adrenal Clock Imposed by Exposure to Constant Light

**DOI:** 10.1371/journal.pone.0042713

**Published:** 2012-08-13

**Authors:** Natalia Mendez, Lorena Abarzua-Catalan, Nelson Vilches, Hugo A. Galdames, Carlos Spichiger, Hans G. Richter, Guillermo J. Valenzuela, Maria Seron-Ferre, Claudia Torres-Farfan

**Affiliations:** 1 Instituto de Anatomía, Histología y Patología, Universidad Austral de Chile, Valdivia, Chile; 2 Programa de Fisiopatología, Instituto de Ciencias Biomédicas (ICBM) Facultad de Medicina, Universidad de Chile, Santiago, Chile; 3 Department of Women's Health, Arrowhead Regional Medical Center, Colton, California, United States of America; Karlsruhe Institute of Technology, Germany

## Abstract

Surprisingly, in our modern 24/7 society, there is scant information on the impact of developmental chronodisruption like the one experienced by shift worker pregnant women on fetal and postnatal physiology. There are important differences between the maternal and fetal circadian systems; for instance, the suprachiasmatic nucleus is the master clock in the mother but not in the fetus. Despite this, several tissues/organs display circadian oscillations in the fetus. Our hypothesis is that the maternal plasma melatonin rhythm drives the fetal circadian system, which in turn relies this information to other fetal tissues through corticosterone rhythmic signaling. The present data show that suppression of the maternal plasma melatonin circadian rhythm, secondary to exposure of pregnant rats to constant light along the second half of gestation, had several effects on fetal development. First, it induced intrauterine growth retardation. Second, in the fetal adrenal *in vivo* it markedly affected the mRNA expression level of clock genes and clock-controlled genes as well as it lowered the content and precluded the rhythm of corticosterone. Third, an altered *in vitro* fetal adrenal response to ACTH of both, corticosterone production and relative expression of clock genes and steroidogenic genes was observed. All these changes were reversed when the mother received a daily dose of melatonin during the subjective night; supporting a role of melatonin on overall fetal development and pointing to it as a ‘time giver’ for the fetal adrenal gland. Thus, the present results collectively support that the maternal circadian rhythm of melatonin is a key signal for the generation and/or synchronization of the circadian rhythms in the fetal adrenal gland. In turn, low levels and lack of a circadian rhythm of fetal corticosterone may be responsible of fetal growth restriction; potentially inducing long term effects in the offspring, possibility that warrants further research.

## Introduction

The environment of mother, baby and child is a key contributor to diseases and conditions that account for approximately one third of the global burden of disease in both developed and developing countries [1]. In this context, chronodisruption (i.e., a significant disturbance of the temporal organization of endocrinology, physiology, metabolism and behavior) along pregnancy has been associated with an increased risk not only of miscarriage, but also preterm delivery and low birth weight [2]–[5]; both strong predictors of chronic disease later in life [6]–[7]. Conceivable, both the maternal and fetal circadian system are the targets of developmental chronodisruption. However, given that the organization of the fetal circadian system is presently unknown, the relationship between maternal photoperiod and fetal and neonatal outcome remains poorly understood.

At variance of the circadian hierarchy in adults, fetal peripheral circadian clocks develop much earlier than the master clock (suprachiasmatic nucleus; SCN). A series of experiments in rats and hamsters demonstrate that within a litter synchrony is lost after maternal SCN ablation; moreover, rhythms persist but are not synchronized in heterozygote fetuses of arrhythmic mothers [8]–[9]. Hence, the tenet is that an endogenous property allows development of circadian rhythmicity in the fetus, nonetheless entrainment of the rhythms is provided by signals controlled by the maternal SCN. Despite the rat SCN not being a functional master clock yet, several fetal organs seem to behave as peripheral oscillators [10]. Among these, the fetal adrenal gland is a key endocrine organ that orchestrates maturational processes central for the transition to extra-uterine life; indeed, a correct maturation of the hypothalamus-pituitary-adrenal axis is essential for normal physiology during adult life [11]. In this context, there is evidence supporting that lack of a circadian rhythm of corticosterone during adult life, in a model of adrenal-specific clockwork disruption, alters the circadian pattern of clock gene expression in other tissues like liver, pancreas and kidney and it somewhat decreases locomotor activity; suggesting that the adrenal clock is required to sustain other peripheral clocks [12]. Recently, we demonstrated that the rat fetal adrenal gland is a strong peripheral circadian clock, at 18 days of gestation, in which corticosterone content follows a circadian rhythm as reported in adult individuals [13]. However, it is presently unknown which maternal signal(s) may generate and/or maintain a fetal corticosterone circadian rhythm.

We hypothesize that the link between the time-keeping systems of the mother and her pups is provided by the maternal circadian rhythm of plasma melatonin. The maternal pineal gland produces melatonin in a circadian fashion, with high plasma levels at nighttime and very low levels at daytime (revised in [10], [14]). Melatonin has the ability to cross all physiological barriers such as the placenta [15]–[16] and the blood-brain barrier [17]. Therefore the fetus, in which the pineal does not synthesize melatonin in several species [18]–[20], is exposed to the maternal melatonin rhythm, and hence indirectly to Light∶Dark (LD) information [21]–[22]. However, exposure to light at night suppresses the nocturnal peak of maternal melatonin depriving the fetus of a LD signal [14], [23]. Accordingly, it has been shown that maternal chronodisruption and particularly melatonin suppression may induce several effects in the rodent circadian system of the fetus and newborn [24]–[25]. In previous work in a non-human primate, we demonstrated that maternal exposure to constant light during the last third of gestation induced precocious maturation of the fetal adrenal, set SCN circadian clock gene expression and resulted in increased plasma cortisol concentrations in the newborn soon after birth [26]–[27], [23]. In fact, recent *in vitro* data obtained in the rat fetal adrenal support that melatonin may set the phase of the clock-controlled gene *Steroidogenic acute regulatory protein* (*StAR*) -which is a key protein in corticosterone synthesis-, as well as of the clock genes *Brain and muscle aryl hydrocarbon receptor nuclear translocator like protein 1* (*Bmal1*) and *Period 2* (*Per2*) [13].

To test our hypothesis, we sought the effects of maternal melatonin on the circadian function of the fetal adrenal gland. To this end, we subjected rats to constant light during the second half of pregnancy with or without melatonin treatment during the subjective night. At 18 days of gestation, the effects of maternal melatonin circadian rhythm suppression and replacement were investigated on: 1) maternal plasma corticosterone and melatonin levels, 2) fetal biometry, 3) circadian and integrated mRNA expression of *Per2*, *Bmal1*, *StAR*, *Melatonin receptor isoform 1* (*Mt1*) and *Early growth response protein 1* (*Egr1*) in the fetal adrenal *in vivo*, 4) fetal adrenal corticosterone content *in vivo*, 5) corticosterone response to ACTH in fetal adrenal culture, and 6) relative mRNA expression of *Per2*, *Bmal1*, *StAR* and *Melanocortin receptor 2* (*MC2R*) in response to ACTH in fetal adrenal culture.

## Materials and Methods

### Animals

Animal handling and care was performed following the NIH Guide for Animal Experimentation Care recommendations and the protocols were approved by the Bioethics Commission from Facultad de Medicina, Universidad de Chile (CBA# 0234).

Timed-pregnant female Sprague-Dawley rats were obtained after mating (embryonic day 0 correspond at the day in which spermatozoa were observed in the vaginal smear) from Bioterio Central, Facultad de Medicina Universidad de Chile. The dams were maintained in a 12∶12 light/dark cycle (lights on at 0700) under controlled temperature (18–20°C) and food and water was available *ad libitum*. At 10 days of gestation dams were weighed and assigned to the following protocols.

### Effects of maternal exposure to constant light on fetal growth and the fetal adrenal clock

A group of 24 dams continued pregnancy in 12∶12 light/dark cycle (control group). Another group of 60 dams were maintained in constant light (LL) until 18 days of gestation. Thirty of these dams received daily melatonin in the drinking water (LL+Mel; 2 µg/ml) at the clock time of the subjective night (1900-0700-h) and the remaining 30 received fresh water (placebo) at the same clock time (LL group). At 18 days of gestation the dams (five mothers per clock time for LL and LL+Mel and 4 mothers per clock time for LD groups) were euthanized with sodium thiopental (150 mg/kg) every 4 hours around the clock; weighed and a blood sample was collected. Fetuses were dissected in sterile conditions and weighed. Dams carried 8 to 14 fetuses evenly distributed in each uterine horn and presented no signs of fetal reabsorption. Dams had a mean increase in maternal weight between 10 and 18 days of gestation of 59.8±6.7 g in LD; 64.56±3.38 g in LL and 66.00±5.61 g in LL+Mel females and weighed 397±10.27 g in LD (n = 24); 402.7±7.624 g in LL (n = 30) and 400.7±14.80 g LL+Mel (n = 30) females at 18 days of gestation. Fetal adrenals were dissected. At each clock time six adrenal glands from fetuses of the same litter were pooled and kept at −20°C for corticosterone measurement. About 15 adrenal glands from the remaining fetuses of a litter were pooled and subjected to RNA extraction in lysis buffer (SV Total RNA Isolation System, Promega, Madison 53711, USA).

### Effects of maternal exposure to constant light on the fetal adrenal gland response to ACTH in culture

In this experiment, we explored the effect of ACTH on corticosterone production in adrenal glands from fetuses from each experimental condition. At 10 days of gestation, 15 dams (5 per group) were allocated to LD, LL and LL+Mel treatment as described above. At 18 days of gestation, dams were euthanized between 2000–2200 hrs under red light, fetuses were dissected and about 100 fetal adrenals were collected per maternal condition. The respective fetal adrenals pools were pre-incubated 6 hrs at at 37°C, 100% humidity, 5% CO2, and 95% air in 15 ml DMEM-F12 (GIBCO, Grand Island, NY, USA). After six hours pre-incubation, 6 fetal adrenals from each pool were incubated in triplicate with 1.5 ml medium alone (control) or with medium containing 10 nM ACTH1-24 (ACTH) for 3 or 6 hrs. At the end of the incubation adrenals and medium were collected and stored for corticosterone and mRNA expression analysis. The ACTH dose was selected based in our previous work [28]. The time in culture was selected to mimic the *in vitro* adrenal response to ACTH reported in adult rodent, in which a significant change in corticosterone production has been detected after 3 hours in culture [29]. In addition, we extended the time in culture to 6 hours in order to measure a putative delay in ACTH response.

### Real-time quantitative RT-PCR (qRT-PCR)

Fetal adrenals totals RNA from in vivo and in vitro experiments were extracted using SV Total RNA Isolation System (Promega) according to the manufacturer's instructions. To assure that genomic DNA was absent in the final reaction, the kit includes a step with DNAse. About 1.0 µg of total RNA was reverse-transcribed at 37°C in a reaction mixture that contained 1.0 µl random primers (50 ng; Promega), 1.0 µl 10 mM dNTPs mix (Promega), 4.0 µl 5× First Strand Buffer (Promega, Madison 53711, USA), 2.0 µl 0.1 M DTT (Promega) and 200 IU M-MLV Reverse Transcriptase (Invitrogen Corp, Carlsbad, CA) in a final volume of 20 µl. The expression levels of the mRNA of *Per2*, *Bmal1*, *Mt1*, *Egr1*, *MC2R*, *StAR* and of the housekeeping genes 18S-rRNA, were measured by real time PCR (qRT-PCR) using the methodology and primers previously described by us [13]. Briefly, the qRT-PCRs were performed in a final volume reaction of 12.5 µl containing 7.5 µl *SYBR^R^ Green Master Mix* (Applied Biosystems, USA) and 0.15 µM of each sense and antisense primers, H_2_O PCR grade and cDNA. The qPCR was carried out in a Applied *Biosystems StepOne™ Real Time PCR System* thermocycler (Applied Biosystems, USA) and consisted of an initial denaturation step at 95°C for 5 min, followed by 50 cycles of 95°C for 30 sec, primer annealing temperature for 30 sec and 72°C for 30 sec. A melting curve analysis was performed on each sample after the final cycle to ensure that a single product was obtained, and agarose gel electrophoresis confirmed that the single PCR product was of the expected size. Relative amounts of all mRNAs were calculated by the comparative ΔΔCt method [30] using the equation 2^−ΔΔCt^.

### Hormone assays

#### Melatonin

*Plasma* Maternal melatonin concentration was measured by double RIA using a commercial kit (MP Biomedical Solon, OH; Melatonin Research RIA Kit Cat# 07L -139102) following the manufacturer's instructions. The inter and intra assay coefficients were less than 10%.

#### Corticosterone

Rat fetal adrenal corticosterone content and maternal corticosterone concentration were measured using the protocol described by Wotus *et al* [31]. Briefly, fetal adrenals were homogenized with 20% ethanol in PBS, then centrifuged at 1200×g at 4°C for 5 min and the supernatants were collected for corticosterone analysis. Fetal adrenal corticosterone content, in vitro corticosterone production and plasma maternal corticosterone concentration were measured by RIA using a commercial kit (Coat-a-Count Rat Corticosterone kit; Siemens Healthcare Diagnostics, Plainfield, IN) following the manufacturer's instructions. The inter and intra assay coefficients were less than 10%.

### Statistical Analysis

Data are expressed as mean ± SEM. The 24-h changes in fetal and maternal corticosterone, maternal melatonin and the values of 2^−ΔΔCt^ for each gene measured were analyzed by one way ANOVA using Newman-Keuls test as a post hoc test. To compare LD, LL and LL+Mel 24-h changes in fetal and maternal corticosterone, maternal melatonin and the values of 2^−ΔΔCt^ for each gene measured were analyzed by two way ANOVA using Bonferroni as a post hoc test. Integrated expression in 24-h for each gene in the fetal adrenal from LD, LL and LL+Mel fetuses was calculated as area under curve (AUC) from a 24-h interval. The effect of ACTH on corticosterone production and gene expression was analyzed by two way ANOVA using Bonferroni as a post hoc test. Statistical analyses were performed using GraphPad Prism software (version 3.02; GraphPad Software Inc., San Diego, CA). Results were considered significant when P<0.05.

## Results

Neither maternal exposure to constant light nor maternal melatonin supplementation shows notorious effect in behavior in the pregnant rat. We did not observe signs of maternal stress, like changes in behavioral conduct (hair loss, presence of stereotyped movements, food intake, etc), or over maternal gain weight between 10–18 days of gestation. In addition we did not observed differences in the number of fetuses and we did not find signs of fetal reabsorption.

**Figure 1:**
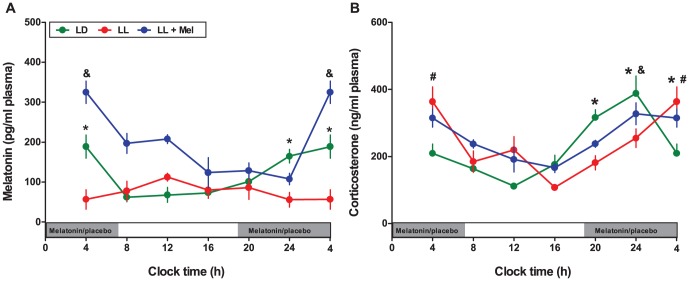
Effect of maternal exposure to constant light and melatonin replacement on daily rhythm of maternal melatonin (A) and corticosterone (B) at day 18 of gestation. Mean ± SEM of corticosterone and melatonin measured in maternal plasma collected every 4 hours from 4 pregnant dams maintained under LD (green symbols), 5 LL dams (*Red symbols*) and 5 LL+Mel dams (*Blue symbols*). *: Different from other time points for LD (P<0.05; ANOVA and Newman-Keuls). **^&^**: Different from other time points for LL+Mel (P<0.05; ANOVA and Newman-Keuls). **^#^**: Different from other time points for LL (P<0.05; ANOVA and Newman-Keuls). The grey bars indicate the clock time of lights off and melatonin replacement or placebo during subjective night.

Constant light effectively suppressed maternal plasma melatonin rhythm with consistently lower values at all clock time points (**Fig. 1A**). In contrast, those pregnant females exposed to constant light but receiving a daily dose of melatonin during the subjective night, presented a circadian rhythm of melatonin with a phase delay of about 4 hours regarding LD mothers acrophase occurring at 05∶56; r^2^ 0.678; for LL+Mel (**Fig. 1A**) and at 02∶27; r^2^ 0.69 for LD mother, respectively.

Despite the absence of a melatonin rhythm, dams exposed to constant light presented similar corticosterone levels (as area under curve in 24-h) than control and LL+Mel mothers (4147±168 for LL; 4393±121 for LL+Mel and 4050±338 for LD mothers; ng/24-h). In addition, a circadian rhythm of corticosterone was present (acrophase at 02∶58; r^2^:0.57) with a phase delay of about 5 hours regarding that of control dams peaking at 22∶36 (**Fig. 1B**). Treatment with melatonin of mothers exposed to constant light slightly advanced the acrophase of corticosterone rhythm (acrophase at 01∶59; r^2^:0.73, **Fig. 1B**) compared to that of control dams.

### Effects of maternal exposure to constant light on fetal growth

**Figure 2:**
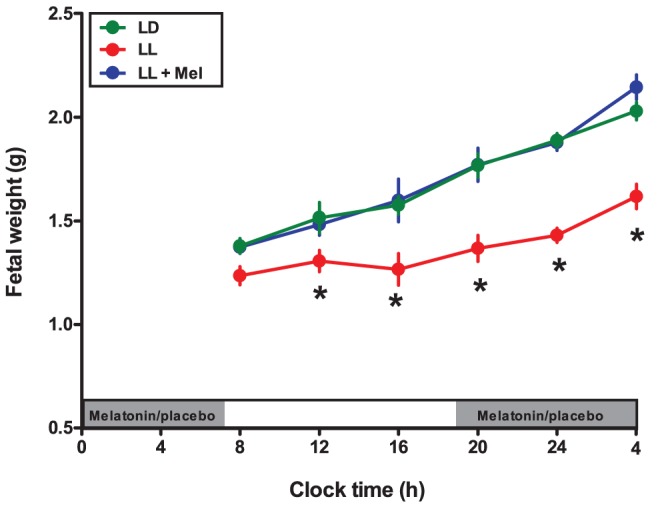
Effect of maternal exposure to constant light and melatonin replacement on fetal weight at different clock times along day 18 of gestation. Mean ± SEM of fetal weight obtained at different clock times from 4 pregnant dams maintained under LD (*Green*), 5 maintained in LL (*Red*) and 5 maintained in LL+Mel (*Blue*). *: Different from LD and LL+Mel (P<0.05; Two way ANOVA and Bonferroni). The grey bars indicate the clock time of lights off and of melatonin replacement or placebo during subjective night.

An important effect of the maternal exposure to constant light was a decrease in the fetal weight regarding that of the fetuses from mothers in LD; this was observed through the 24-h at 18 days of gestation (**Fig. 2**). This growth retardation secondary to maternal melatonin suppression was also observed in other biometric parameters, such as crown to rump length, bi-parietal diameter and femur length (**Table 1**). Maternal melatonin replacement (LL+Mel) restored fetal body weight as well as crown to rump length, bi-parietal diameter and femur length to values similar that those found in LD fetuses (**Fig. 2**) (**Table 1**).

**Table 1:** Effects of maternal exposure to constant light and maternal melatonin replacement on fetal biometry at day 18 of gestation (mean ± SEM).

24-mean	LD (n = 55)	LL (n = 63)	LL + Mel (n = 57)
**Litter size (number)**	13.92±0.36	13.89±0.33	13.64±0.31
**Body weight (g)**	1.69±0.13	1.37±0.05*	1.71±0.21
**Crown to rump length (mm)**	25.1±0.20	22.0±0.42*	25.8±0.2
**Femur length (mm)**	6.38±0.11	4.17±0.11*	5.27±0.17
**Biparietal diameter (mm)**	5.86±0.04	5.71±0.04*	5.88±0.03

***LD***, mothers under light-dark cycle 12∶12. ***LL***, mothers under constant lights between 10 and 18 days of gestation. ***LL+Mel***, Mothers under LL receiving a daily oral dose of melatonin. ***n***, number of animals.

* : Different from LD and LL+Mel (P<0.05, ANOVA and Newman-Keuls).

### Effects of maternal exposure to constant light on the fetal adrenal clock

**Figure 3:**
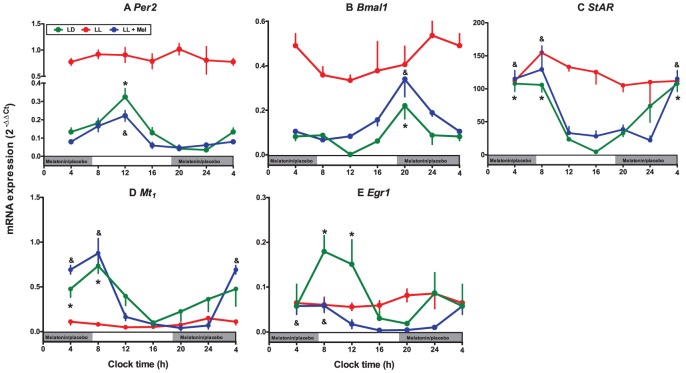
Effect of maternal exposure to constant light and maternal melatonin replacement on oscillatory transcription of *Per2* (A), *Bmal1* (B), *StAR* (C), *Mt1* (D) and *Egr1* (E) in rat fetal adrenal gland at day 18 of gestation. Mean ± SEM of 2^−ΔΔCt^. Fetal adrenal glands were collected every 4 hours from fetuses obtained from pregnant dams under LD (green symbols), LL (*Red symbols*) and LL+Mel (*Blue symbols*). *: Different from other time points for LD (P<0.05; ANOVA and Newman-Keuls). **^&^**: Different from other time points for LL+Mel (P<0.05; ANOVA and Newman-Keuls). LL samples are different from LL+Mel and LD samples (P<0.05; Two way ANOVA and Bonferroni). The grey bars indicate the clock time of lights off and of melatonin replacement or placebo during subjective night.

The fetal adrenal circadian clock was markedly affected by maternal exposure to constant light. In fact, the clock transcripts *Per2* and *Bmal1* did not oscillate at all (**Fig. 3A–B**); which was accompanied by a lack of oscillation in the transcription of *StAR*, *Mt1* and *Egr1* (**Fig. 3 C–E**). Notably, in the fetal adrenal gland, maternal melatonin replacement (LL+Mel) restored the circadian rhythm of all transcripts measured (*Per2*, *Bmal1*, *Mt1 StAR* and *Erg1*; **Fig. 3 A–B**) with a slight phase advanced compared to that found in the fetal adrenal gland from LD fetuses (**Fig. 3 A–E,** **Table 2**). Thus, expression of *Per2* and *Bmal1* in the adrenal of LL+Mel fetuses fitted theoretical cosine functions with a maximum at 12∶42 for *Per2* and 20∶56 min for *Bmal1* (**Table 2**); i.e., a delay between them of about 8 h, and with a maximum at 06∶36 for StAR, 06∶15 for Mt1 and 5∶21 for *Erg1*. Comparing the integrated expression of *Per2*, *Bmal1*, *StAR*, *Mt1* and *Egr1* (AUC of 2^−ΔΔCt^) in the adrenal gland from LD, LL and LL+Mel fetuses, we observed that the adrenal gland from LL fetuses presented a lower expression of Mt1 versus both, LD and LL+Mel condition (**Table 2**). In contrast, maternal melatonin suppression (LL) increased the expression of *Per2*, *Bmal1* and *StAR* (**Table 2**), situation that was reversed in the adrenals from fetuses whose mothers received melatonin replacement. Interestingly, constant light did not modify the integrated expression of Egr1, but maternal melatonin replacement induced a decrease in *Egr1* expression in the fetal adrenal.

**Table 2:** Effect of maternal exposure to constant light and maternal melatonin replacement on the circadian rhythms and integrated mRNA expression of *Bmal1*, *Per2*, *StAR*, *Mt1* and *Egr1* in the rat fetal adrenal gland.

	LD			LL			LL+Mel		
	φ (hours)	r^2^	AUC (2^−ΔΔCt^/24 h)	φ (hours)	r^2^	AUC (2^−ΔΔCt^/24 h)	φ (hours)	r^2^	AUC (2^−ΔΔCt^/24 h)
***Per2***	12.7±0.3	0.71	1.8±0.22	**-**	**-**	8.0±0.5*	9.7±0.2#	0.72	1.7±0.3
***Bmal1***	22.1±0.6	0.64	3.0±0.3	**-**	**-**	17.7±1.2*	22.9±0.5	0.70	2.3±0.2
***StAR***	6.2±0.4	0.66	1031±107	**-**	**-**	2517±154*	6.6±0.5	0.64	1191±129
***Mt1***	7.3±0.3	0.71	2.1±0.2	**-**	**-**	1.6±0.1*	6.3±0.2	0.70	0.6±0.1
***Egr1***	9.4±0.4	0.62	7.5±0.7	**-**	**-**	1.6±0.2*	5.3±0.5#	0.62	6.2±0.9

***LD***, mothers under light-dark cycle 12∶12 (n = 4/clock time). ***LL***, mothers under constant lights between 10 and 18 days of gestation (n = 5/clock time). ***LL+Mel***, Mothers under LL receiving a daily oral dose of melatonin (n = 5/clock time).

* : Different from LD and LL+Mel (P<0.05, ANOVA and Newman-Keuls).

# : Different from LD (Student t test).

**Figure 4:**
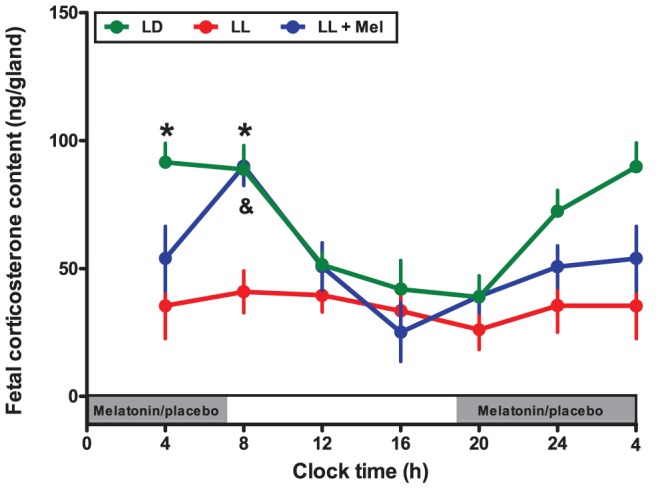
Effect of maternal exposure to constant light and maternal melatonin replacement on the daily rhythm of corticosterone content in the rat fetal adrenal gland at day 18 of gestation. Mean ± SEM of fetal corticosterone content. Fetal adrenal glands were collected every 4 hours from fetuses obtained from pregnant dams under LD (green symbols), LL (*Red symbols*) and LL+Mel (*Blue symbols*). *: Different from other time points for LD (P<0.05; ANOVA and Newman-Keuls). **^&^**: Different from other time points for LL+Mel (P<0.05; ANOVA and Newman-Keuls). LL samples are different from LL+Mel and LD samples (P<0.05; Two way ANOVA and Bonferroni). The grey bars indicate the clock time of lights off and of melatonin replacement or placebo during subjective night.

In addition, we found that maternal exposure to constant light decreased fetal adrenal corticosterone content and abolished the circadian rhythm (**Fig. 4**). However, maternal melatonin replacement (LL+Mel) fully restored the fetal adrenal rhythm of corticosterone content to similar concentration than those found in control condition, with a slight difference in the acrophase of the rhythm, that was found at 08∶07 (r^2^:0.574), about 2 hours apart that those found in control conditions at 06∶13 (r^2^: 0.79; **Figure 4**).

### Effects of maternal exposure to constant light on the fetal adrenal gland response to ACTH in culture

**Figure 5:**
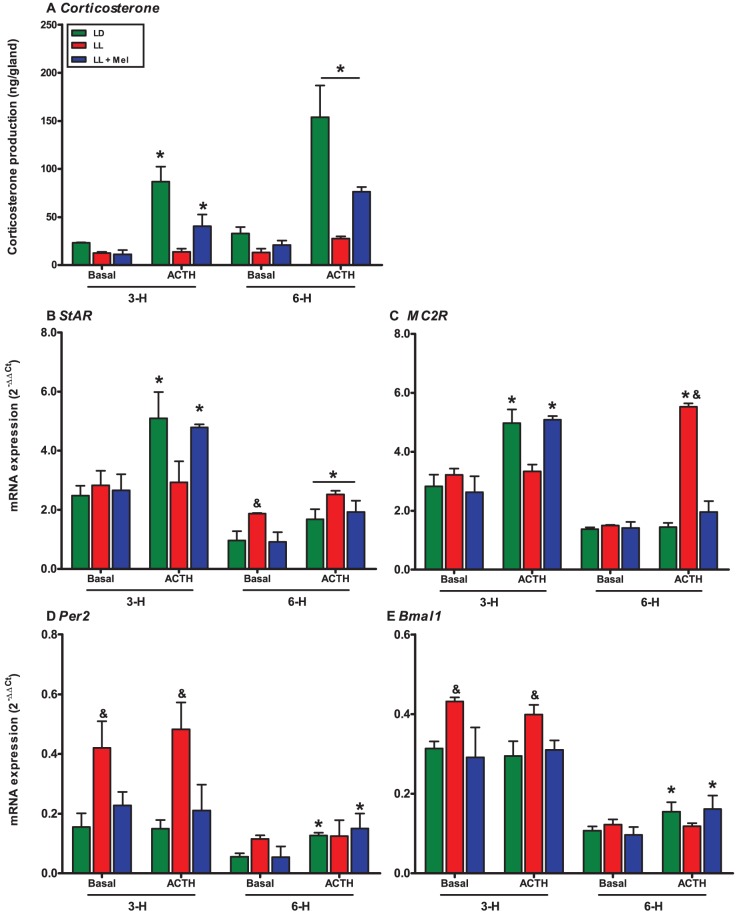
Effect of maternal exposure to constant light and maternal melatonin replacement on corticosterone production (a), *StAR* (b), *MC2R* (c), *Per2* (d) and *Bmal1* (e) in response to ACTH in fetal adrenal gland in culture. Mean ± SE for fetal corticosterone production and for 2^−ΔΔCt^ in culture at 3 and 6 hours. Adrenal glands were collected between 2000 and 2200 hours from fetuses coming from pregnant dams under LD (*Green bars*), LL (*Red bars*) and LL+Mel (*Blue bars*), pre-incubated for 6 hrs and then with ACTH 10 nM along either 3 or 6 hours. *: different to basal production or expression at the same time in culture (P<0.05; Two way ANOVA and Bonferroni). &: Different to LD and LL+Mel (P<0.05; Two way ANOVA and Bonferroni).

Basal corticosterone production was lower in adrenal explants from LL fetuses than in explants from control fetuses at 3 and 6 hours of culture. In addition, there were differences in the corticosterone response to ACTH. We observed that ACTH elicited an about 4-fold increases of corticosterone production in LD fetal adrenals after 3 hours in culture, that increased further meanwhile after 6 hours in culture whilst a minor response was observed in LL adrenals only at 6 hours of culture. (**Fig. 5A**). Maternal melatonin replacement partially restored the early corticosterone response to ACTH, but to lower levels than in control 6 hours after ACTH treatment (**Fig. 5A**).

A similar finding was detected for *StAR* and *MC2R* expression in which ACTH stimulate their expression in LD and LL+Mel fetal adrenals at both hours in culture studies (**Fig. 5 B–C**), whilst in adrenal from LL fetuses we observed a significant response only after 6 hours in culture with ACTH. In addition, we observed that ACTH stimulates *Per2* and *Bmal1* expression in fetal adrenal gland after 6 hours in culture, response that was absent in fetal adrenals from LL condition (**Fig. 5 D–E**).

## Discussion

The present data show that exposure of pregnant rats to constant light along the second half of gestation effectively suppressed maternal plasma melatonin rhythm, whilst it shifted the phase of the rhythm but did not change the integrated levels of maternal circulating corticosterone. Maternal melatonin suppression had effects on fetal development and in fetal adrenal function. First, it induced intrauterine growth retardation. Second, in the fetal adrenal *in vivo* it markedly affected the mRNA expression level of *Per2*, *Bmal1*, *StAR*, *Mt1* and *Egr1* as well as it lowered corticosterone content. Third, using a fetal adrenal culture paradigm, we were able to establish that maternal exposure to constant light modified the *in vitro* response to ACTH of both, corticosterone production and relative mRNA expression of *StAR*, *MC2R*, *Egr1*, *Per2* and *Bmal1*. The changes in intrauterine growth and fetal adrenal function, secondary to maternal exposure to constant light, were reversed when the mother received a daily dose of melatonin during the subjective night. Taken together, these results are in agreement with our recent work [10], [13], showing that the rat fetal adrenal is a functional peripheral oscillator. The present evidence supports a role of maternal melatonin on overall fetal development and for the rat fetal adrenal gland.

Exposure of pregnant dams to constant light during the second half of gestation effectively suppressed the maternal melatonin rhythm as has been shown by us and others [32], [23]. Intuitively, this treatment might be seen as a powerful maternal stressor, an indeed other authors have shown that it disrupts the rhythm of locomotor activity in the pregnant rat [33]. However, we did not find changes on maternal corticosterone production and other established behavioral variables related with maternal stress. Thus, in the present rat model, maternal chronodisruption and the ensuing melatonin rhythm suppression imposed by exposure to constant light may induce direct effects in fetal physiology, rather than indirectly through the maternal stress axis. However, we can not discard effects of the treatment over other maternal circadian rhythms not tested here. Indeed, temperature is a circadian rhythm modified by exposure to constant light in non pregnant animals [34]; however, evidence of our group support that a maternal circadian rhythm of temperature is present in pregnant capuchin monkey exposed to constant light, which offspring presented free-running circadian rhythm of temperature [35]. It is unknown whether this is the situation in the rat and undoubtedly more studies are required to determine the whole impact of maternal circadian rhythms on fetal development.

In our model of maternal chronodisruption, we observed significant differences in fetal biometric parameters under constant light relative to control (LD) conditions. Interestingly, when we monitored fetal growth every 4-h along day 18 of gestation, there was a slower growth rate in the fetuses from mothers kept under constant light relative to control (LD) conditions. The detection of growth retardation inside such a short time window is not surprising, considering that in the rat fetal growth is exponential and maximal by the end of gestation [36]. Furthermore, comparable intrauterine growth restriction, together with placental edema, fibrin accumulation and leukocyte infiltration have been reported in rat pregnancy under constant light [37], whilst maternal pinealectomy induced an increase in pup malformations with low weight at birth [38]. Indeed, our biometric results in the rat are consistent with epidemiological studies of human pregnancy subjected to chronodisruption pointing to an increased risk of miscarriage, preterm delivery and particularly low birth weight [2]–[5]. The present finding that fetal growth restriction imposed by light regime alteration is fully reversed by maternal melatonin administration during subjective night, might be relevant to shift working pregnant women.

Together with the decrease in growth rate and body weight, we found marked effects of maternal chronodisruption on the fetal adrenal gland. Our results indicate that the circadian pattern of expression in antiphase was lost for the core clock genes *Per2* and *Bmal1*, concurrently with lack of oscillatory transcription of the clock-controlled genes *StAR*, *Mt1* and *Egr1* in fetuses from mothers exposed to constant light relative to either control (LD) or constant light plus melatonin conditions. In addition, maternal exposure to constant light suppressed the circadian rhythm of corticosterone content and most probably reduced circulating fetal corticosterone as well. However, we can not discard that the present observations may be due to a desynchronized instead of arrhythmic litter, as reported by others in the offspring gestated in arrhythmic mothers; which displayed circadian rhythms of temperature, corticosterone and gene expression which were desynchronized between the litters [9], [39]–[40].

Regulation of glucocorticoids synthesis and release is complex involving several converging regulatory signals in the adult [41], whereas even less is known for this key adrenal function in the fetus. Thus, it is not easy to explain why fetal adrenal corticosterone content was reduced in the group coming from LL mothers. To shed light on this issue, for the first time, we measured *in vitro* fetal adrenal corticosterone production and the transcription rate of several genes previously reported to be induced by ACTH in other species [42]–[44]. Although this experimental design is limited in terms of unveiling a mechanism, we found that the fetal adrenal responsiveness to ACTH was modified by maternal exposure to constant light but it was restored by maternal melatonin treatment. In adrenals from control fetuses, we found that in vitro treatment with ACTH induces a prompt increase in fetal adrenal corticosterone production and of *StAR* and *MC2R* expression. Continuing the treatment with ACTH results in a further increase in corticosterone production accompanied of increased expression of *Per2* and *Bmal1*. No increase in corticosterone was observed in LL fetal adrenals and the response was restored in LL+Mel fetal adrenals. These *in vitro* data are consistent with the *in vivo* data and suggest a reduced responsiveness to ACTH of the adrenal glands of fetuses whose mothers were exposed to constant light. In these adrenals, ACTH induced a late increase in *MC2R* and *StAR*, but it had no effect on *Per2* and *Bmal1* expression. It is important to keep in mind that lack of adrenal response of both, corticosterone production and gene transcription at either 3 or 6 hours of exposure to ACTH, does not rule out that the given response might occur using a longer time of incubation. Nonetheless, we speculate that disruption of the adrenal circadian clock might explain lack of ACTH responsiveness. This possibility is supported by the observation that clock genes were arrhythmic in the *in vivo* adrenal of fetuses from LL mothers and by previous reports showing that integrity of the adult adrenal clockwork machinery is crucial for ACTH response [42], [29]. Although the intimate adrenal molecular link between clock genes oscillatory expression and ACTH actions remains unknown, the present results are in line with the hypothesis that the integrity of the circadian system is key for fetal adrenal physiology and ACTH response, as has been reported in adult species [42]. A possibility is that maternal melatonin suppression interfered with adrenal maturation, given that in the rat it has been reported that adrenal corticosterone content declines at the end of gestation reaching low values immediately after birth [31]. Besides, there is evidence that corticosterone response to stress is dumped in the rat newborn [45]; supporting that a decreased response to ACTH may be physiologically appropriate in the perinatal period in the rat. If this is true, then the adrenal's functional status may change along antenatal maturation and, thereby, the observed lack of (or delayed) response to ACTH of *in vitro* adrenals from LL mothers could be a consequence of an accelerated maturation of the fetal adrenal gland (i.e., closer to the newborn's adrenal). This possibility is clearly in line with our previous findings in the capuchin monkey, in which maternal melatonin suppression induced early maturation of the fetal adrenal gland [26]. At any rate, exposure to maternal melatonin seems to be necessary for the proper response to ACTH of the rat fetal adrenal cortex at 18 days of gestation.

A key finding of the present investigation is that all the effects of maternal melatonin circadian rhythm suppression on fetal growth and adrenal function were prevented by maternal melatonin treatment during subjective night. This strongly supports that maternal melatonin rhythm is a paramount signal for normal fetal physiology. The mechanism behind such protective effects of melatonin replacement could rely on direct effects on the fetal adrenal clock. In this context, we recently reported not only that the expression of melatonin receptors displays a circadian rhythm in the rat fetal adrenal at 18 days of gestation, but also that the adrenal seems to be a strong peripheral clock in the fetus, potentially subjected to direct regulation by maternal melatonin [13], [46]. The present results are in full agreement with these previous studies and, in addition, show that lack of melatonin in the fetal circulation (secondary to maternal exposure to constant light) leads to profound alterations of fetal development and adrenal function. The consequences for the fetus are unknown, however, it has been reported that a maternal diet low in sodium, induced low levels of fetal glucocorticoids and intrauterine growth retardation [47]. During fetal life, glucocorticoids are key in the transition to extrauterine life and its receptor is widely distributed in the fetal organs. For instance, it is well established that glucocorticoids play an essential role on maturation of the lung and other fetal organs [48]. In addition, in the adult, the adrenal gland is a strategic peripheral oscillator influencing a number of physiological processes (metabolic, cardiovascular, immune and neural functions) through glucocorticoid circadian oscillation; most probably acting as an entrainment signal for other peripheral clocks [12], [49]–[50]. Peripheral circadian clocks are present in fetal tissues [10]. Thus, suppression of maternal melatonin by exposure to constant light may have a wide impact on fetal organs; most probably by reducing circulating fetal corticosterone and abolishing the fetal corticosterone rhythm.

Regarding the potential relevance of the present findings to human health, it must be kept in mind that among the perinatal environmental risk factors imposed by modern 24/7 society, shift work either ‘rotating’ or ‘permanent’ affects about one fifth of the workforce, including pregnant woman; which means that even a modest detrimental impact on health may have important public health implications [51]. Recently, Varcoe and colleagues demonstrated that maternal exposure to chronic phase shifts of the photoperiod (mimicking a shift work schedule) during pregnancy induced glucose intolerance and insulin resistance in the rat at later postnatal stages [52]. In this context, our general idea is that maternal chronodisruption (i.e., melatonin suppression) is a somewhat neglected deleterious signal for the fetus which might carry on into adulthood as abnormal physiological traits. This possibility needs to be seriously taken into account, as there is a whole body of evidence of a maternal deleterious environment programming susceptibility to diseases that appear in adult life like diabetes, hypertension, obesity and metabolic syndrome [7], [53]–[55].

In conclusion, the present results collectively support that the maternal circadian rhythm of melatonin is a key signal for the generation and/or synchronization of the circadian rhythms in the fetal adrenal gland. In turn, low levels and lack of a circadian rhythm of fetal corticosterone may be responsible of fetal growth restriction; potentially inducing long term effects in the offspring, possibility that warrants further research.
